# Influence of a portable audio-biofeedback device on structural properties of postural sway

**DOI:** 10.1186/1743-0003-2-13

**Published:** 2005-05-31

**Authors:** Marco Dozza, Lorenzo Chiari, Becky Chan, Laura Rocchi, Fay B Horak, Angelo Cappello

**Affiliations:** 1 Department of Electronics, Computer Science, and Systems, University of Bologna, Bologna, Italy; 2 Neurological Science Institute, Oregon Health & Science University, Portland (OR), USA

## Abstract

**Background:**

Good balance depends on accurate and adequate information from the senses. One way to substitute missing sensory information for balance is with biofeedback technology. We previously reported that audio-biofeedback (ABF) has beneficial effects in subjects with profound vestibular loss, since it significantly reduces body sway in quiet standing tasks.

**Methods:**

In this paper, we present the effects of a portable prototype of an ABF system on healthy subjects' upright stance postural stability, in conditions of limited and unreliable sensory information. Stabilogram diffusion analysis, combined with traditional center of pressure analysis and surface electromyography, were applied to the analysis of quiet standing tasks on a Temper foam surface with eyes closed.

**Results:**

These analyses provided new evidence that ABF may be used to treat postural instability. In fact, the results of the stabilogram diffusion analysis suggest that ABF increased the amount of feedback control exerted by the brain for maintaining balance. The resulting increase in postural stability was not at the expense of leg muscular activity, which remained almost unchanged.

**Conclusion:**

Examination of the SDA and the EMG activity supported the hypothesis that ABF does not induce an increased stiffness (and hence more co-activation) in leg muscles, but rather helps the brain to actively change to a more feedback-based control activity over standing posture.

## Background

Maintaining balance is a complex task accomplished by the brain through the fusion and interpretation of sensory information. When sensory information from vestibular, somatosensory, and visual systems [1-3] are not accurate and/or adequate, balance will be compromised. Although, in many cases, the loss of peripheral sensory information is not curable or reversible, the brain can compensate for the loss of sensory information by relying more on the other sensory channels [4,5].

The purpose of biofeedback (BF) systems for postural control is to provide additional sensory information about body equilibrium to the brain [6]. In the last few years, different sensors, encoding algorithms, and information restitution devices have been combined to develop promising BF systems for postural control [7-9]. The major design goals were focused on portability, usability, economy, and effectiveness in balance improvements [8,10-12].

The development of these BF systems has been facilitated by the availability of lightweight, miniaturized, and economical sensors such as accelerometers, inclinometers, and gyroscopes [13]. The use of these sensors makes BF devices inexpensive, unsusceptible to shadowing effect, and not limited in the measurement field, in contrast to dynamometric platforms and motion analysis systems, which are commonly used in laboratory settings [14,15]. In addition, due to their size and weight, these sensors can measure body segment movement without hindering natural motor execution.

More detail is needed in understanding how biofeedback information interacts with the brain or, from a neuroscience perspective, how the brain uses artificial BF information and combines it with natural sensory information. We believe that understanding this interaction is fundamental for further developing effective BF systems.

An interesting analysis in the understanding of how the brain may use BF information for postural control was proposed by Collins and De Luca [16]. These authors developed a statistical-biomechanics method for analyzing force platform data recorded during quiet standing, called stabilogram diffusion analysis (SDA). SDA was applied to center of pressure (COP) data and it disclosed that COP tends to drift away from a relative equilibrium point over short-term observation intervals (less than 1-second long), whereas COP tends to return to a relative equilibrium point over long-term observation intervals. These results took Collins and De Luca to suggest that the motion of the COP is not purely random, and that SDA may be able to give insight on the amount of open-loop and closed-loop postural control applied by the central nervous system for maintaining balance [17]. SDA was used in several contexts, e.g. to evaluate the effect of spaceflight [18], visual input [19,20], and age-related changes [21,22] on postural stability. Chiari el al [20] developed and validated a new nonlinear model for extracting parameters from SDA diagrams, reducing from 6 to 2 the number of the parameters used to characterize the structural properties of COP. Rocchi et al. [23] found that these new parameters may be useful adjuncts to evaluate postural control strategies in patients with Parkinson's disease and may allow the comparison of different deep brain stimulation electrode sites based on their effect on structural properties of the COP.

In this paper, we investigate the effect on postural stability of a portable, accelerometry-based, audio biofeedback (ABF) system recently developed by the authors [9]. Standing with eyes closed on Temper™ foam will be used to evaluate the effects of artificial auditory cues to enhance the limited (from the eyes) and unreliable (from the feet) natural sensory information. Measurements include COP recorded by a force platform under the feet, trunk acceleration measured by the ABF sensors, and EMG signals from the leg muscles. SDA according to [20], traditional COP analysis [24], and muscle activation analysis according to [25] were performed in order to evaluate the effect of ABF on healthy young subject's upright posture.

These analyses were aimed to answer two questions: (1) do structural properties of postural sway change with ABF? And, if so, (2) in which way will this help in understanding the mechanisms underlying ABF efficacy and in improving the design of a rehabilitation strategy for balance disorders?

In this paper, we present evidence that supports the hypothesis that ABF does not induce a purely biomechanical increase in stiffness (and hence more co-activation) in the leg muscles, but rather ABF helps the brain actively adapt its control activity over standing posture.

## Methods

### Participants

Eight healthy subjects participated in this study (5 males and 3 females, aged 23.5 ± 3.0 yrs, range 21–28 yrs). All participants were free from any neurological, orthopaedic, hearing, or vestibular disorder. Informed consent form was obtained from each subject. The form was prepared in accordance with the Oregon Health and Science University Ethical Committee and respected the declaration of Helsinky, 1964.

### Apparatus and procedure

**Figure 1:**
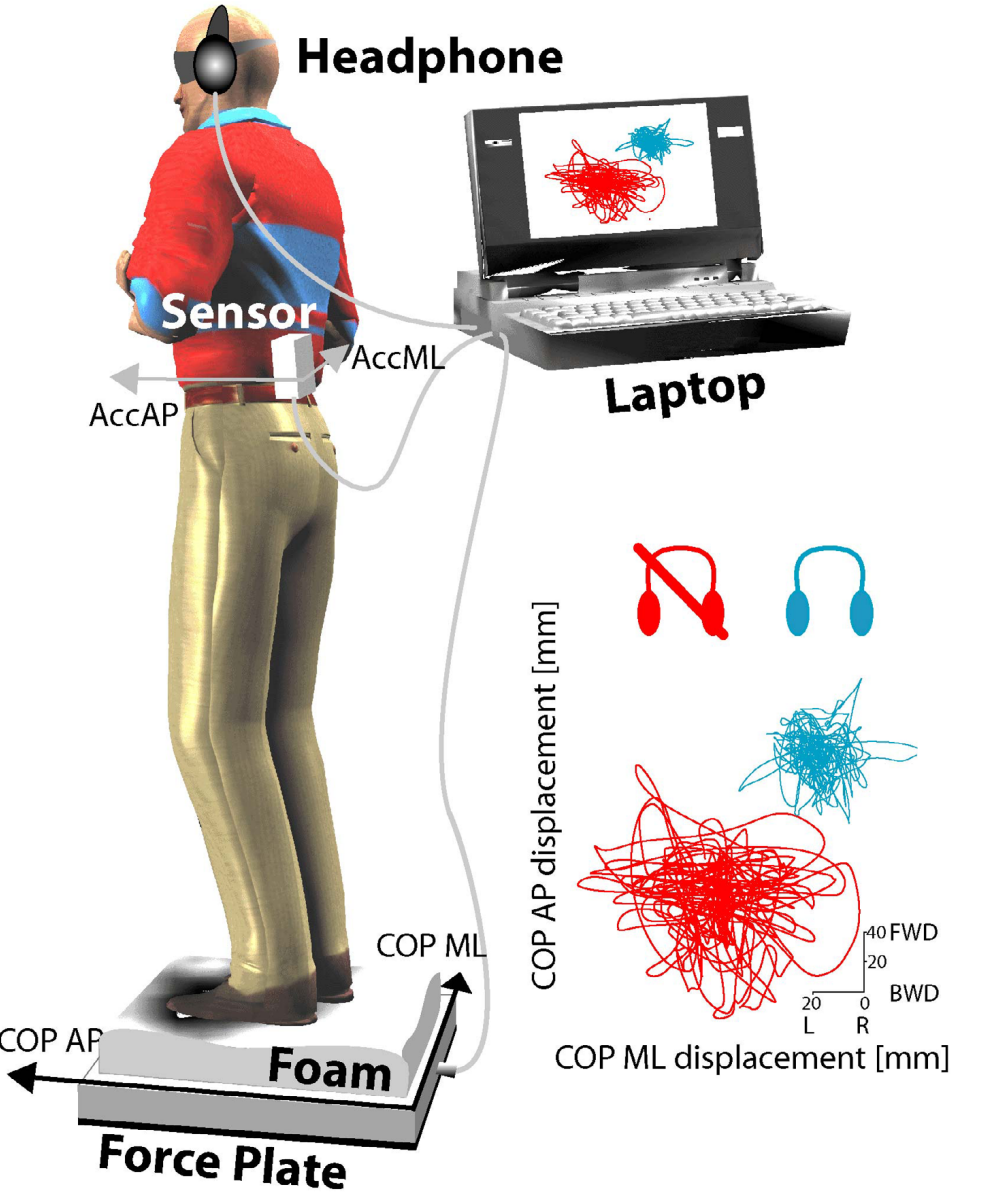
**ABF system device and protocol**. The ABF consisted of (1) a sensor mounted on the trunk that measured accelerations along AP and ML axes, (2) a laptop acquiring acceleration from the sensor and processing the ABF sound, (3) a pair of headphones the subject wore for listening to the sound. In this figure is also shown the protocol in which a healthy subject is standing with eyes closed on a temper foam pad placed on a force plate. At the bottom right of the figure are statokinesigrams in condition with and without ABF from a representative subject.

Subjects performed 10, 60-second trials standing with eyes closed on Temper™, 4"-thick foam. COP displacement was recorded with an AMTI OR6-6 force plate. An ABF system [9] was used to provide subjects with additional balance information related to trunk acceleration. The ABF system used a sensor, based on 2-D accelerometers (Analog Device ADXL203) mounted on the subject's back (L5), to create an audio stereo sound representing the acceleration sensed along the anterior-posterior (AP) and the medial-lateral (ML) direction. A laptop, Toshiba Celeron 2.3 GHz, was dedicated to convert the accelerations into stereo sounds. Commercial headphones were used by the subjects to listen to the ABF sound. The ABF system is described in detail in [9] and illustrated in Figure 1. In short, the stereo sound provided by the ABF system consisted of two sine waves, one for the left ear channel and one for the right ear channel. Pitch, volume and left/right balance of the stereo sound were modulated to represent the 2-D acceleration information. Specifically, when the subject swayed forward, and consequently the acceleration increased in the anterior direction, the sound got louder in volume and higher in pitch. When the subject swayed backward, and consequently the acceleration increased in the posterior direction, the sound got louder in volume and lower in pitch. When the subject moved right and, consequently, the acceleration increased in the right direction, the sound got louder in the right ear channel and lower in the left one. When the subject moved left, and consequently the acceleration increased in the left direction, the sound got louder in the left ear channel and lower in the right one. The sound dynamics was optimized for each trial by taking as a reference the first 10-second recordings of each trial. The equations used for the pitch, volume, and left/right balance modulation can be found in [9]. Each subject was instructed to maintain balance during the trials by taking advantage of the ABF information, when available. Five trials with ABF and 5 trials without ABF were performed in randomized order by each subject. Before the experimental session, the subjects were instructed on how ABF codes trunk acceleration into sound, and performed free-movement trials until they felt confident in performing the full experiment.

### Data recording

For each standing trial, ground reaction forces and torques were recorded from the force plate with a 100-Hz sampling frequency. COP displacement was computed offline from the force plate data after applying a 10-Hz cut-off, zero phase, low-pass Butterworth filter. Accelerations from the trunk along AP and ML direction were collected with a 100 Hz sampling frequency. EMG was recorded from right leg muscles, Tibialis (TI), Soleus (SO), and Gastrocnemius (GA) with two surface electrodes fixed about 6–8 cm apart along the length of each muscle belly; the ground electrode was fixed on a bony area of the right Hallux. The EMG signals were acquired with a 100-Hz sampling frequency, amplified 20000 times, band-pass filtered (71-2652 Hz), integrated with a 6th order Butterworth low-pass filter with a cut-off of 100 Hz (National Semiconductor MF6-100), and full-wave rectified.

### Data analysis

From AP COP data, the root mean square distance (COP-RMS) and the frequency comprising the 95% of the power (F95%) were extracted according to Prieto et al. [24].

From the acceleration sensed at trunk level along AP direction we computed the root mean square value (Acc-RMS).

In addition, two stochastic parameters were included in the analyses. These parameters characterize a previously developed model that describes with continuity the transition among the different scaling regimes found in the COP time series [20] The model is described by the following equation:

V(Δt) = K Δt^2H(Δt)^

where V(Δt) is the variance of COP displacement, computed at time-lag Δt, and H is the scaling exponent, also called Hurst exponent. This is assumed to follow a sigmoid law in the time interval (Δt):



In this way, the features extracted from COP data are the following (see [20] for more details):

- K is an estimate of the diffusion coefficient of the random process obtained by sampling the COP time series at the sampling frequency 1/ΔTc.

- ΔTc represents the time-lag at which the real process corresponds to a purely random behavior, and where it switches from a persistent (positively correlated, and hence interpreted in terms of feed-forward control) to an anti-persistent (negatively correlated, and hence interpreted in terms of feedback control) behavior [16].

Mean muscular activity was calculated from the full wave rectified EMG of each muscle. Muscle activity was expressed as percentage of the maximal recorded activity for each muscle in each subject. This procedure allowed a reliable comparison of muscle activity between-subjects. The EMG signals were further processed applying a zero phase, low pass-filter with a 2 Hz cut-off in order to obtain tension curves according to Olney and Winter [25]. These tension curves were cross-correlated to determine the amount of co-activation between the muscles recorded.

### Statistical analysis

Paired T-tests were performed to determine the effect of ABF on the different parameters extracted from COP, acceleration and EMG data collected. The threshold for statistical significance was set to p = 0.05.

## Results

### Subjects' confidence and comfort

All participants reported ABF sound was comfortable and its way of representing the information was intuitive. In fact, none of the subjects needed more than two, free-movement trials before feeling ready to start the experiment.

### Subjects' sway

**Figure 2:**
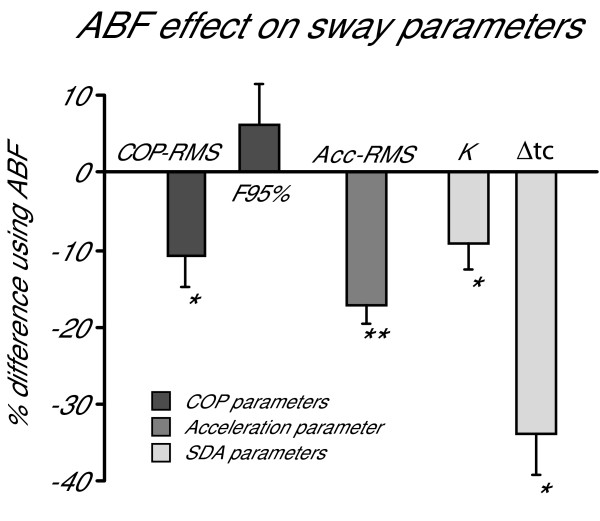
**Effect of ABF on sway**. The percent change of using ABF on the sway parameters is shown. COP-RMS and F95% were extracted from the AP COP displacement according to [24]. Acc-RMS was extracted from AP acceleration recorded at trunk level (L5). K and ΔTc were derived by applying the method proposed by Chiari et al. [20] on the SDA diagrams [16]. Asterisks indicate statistical significance: * p < 0.05 and ** p < 0.01. The reductions of K, COP-RMS and Acc-RMS are a consistent evidence of the reduction of sway amplitude shown by the subject using ABF. The increasing of F95% suggests that the postural control applied by the CNS when ABF is available was increased. The reduction of ΔTc suggests a major active closed-loop postural control exercised by the CNS.

ABF significantly influenced subjects' balance on the foam. The percentage change induced by ABF on all sway parameters, either measured at the trunk level with the accelerometer or at the feet level with the force platform, is shown in Figure 2. Figure 2 also reports significance levels of the parameter changes occurred while using the ABF. The general results shown in Figure 2 are specified in detail in the following.

### Center of Pressure analysis

Center of pressure displacement in the AP direction was significantly influenced by ABF. T-test results revealed significant effects of ABF on COP-RMS (p = 0.015). This effect is shown by a consistent reduction of COP-RMS for 7 out of 8 subjects as shown in Table 1 (column 7). Average reduction of COP-RMS was 10.7%. Columns 1 and 4 of Table 1 also show the subject-by-subject values of COP-RMS without and with ABF, respectively. The last three subjects (#6, #7, #8) were females and showed smaller COP-RMS, as expected considering their smaller heights [26].

F95% increased with ABF for 7 out of 8 subjects (Table 1, column 8) but this result was not significant (p = 0.42). The values of F95% are also reported for each subject in both conditions (Table 1, columns 2 and 5). Average increase of F95% due to ABF was 6.2% as shown in Figure 2.

**Figure 3:**
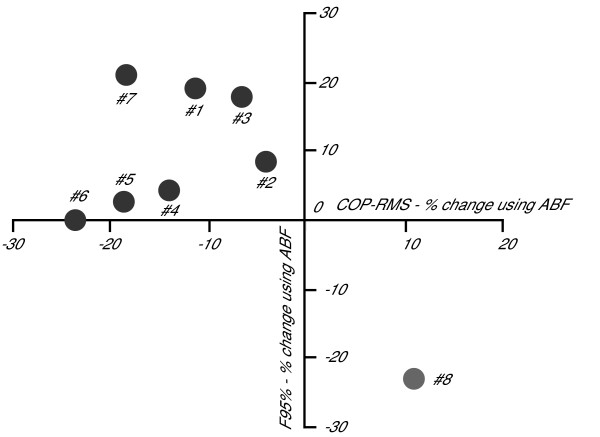
**Antithetic behaviour of subject #8**. COP-RMS percentage change using ABF is reported on the horizontal axis and F95% percentage change using ABF is reported on the vertical axis. The values of each subject from Table 1 are plotted. Subject #8 clearly behaves antithetically to the other subjects.

It is worth noting that subject #8 behaved as an outlier (Figure 3), compared to the other subjects since she was the only one who showed opposite changes in COP-RMS and F95% while using ABF. Performing the T-Tests, after eliminating this outlier, increased the significance of using ABF on COP-RMS and on F95% (p = 0.002 and p = 0.02, respectively). These results better match the results already published in [9]. The outlying behavior of subject #8 will be investigated further in the discussion.

**Table 1:** ABF effect on sway parameters Parameters. COP-RMS, F95%, and Acc-RMS are reported, subject-by-subject, for trials with and without ABF. Percentage differences between these two conditions are also reported. Standard deviations are indicated in parenthesis.

	COP-RMS (NO – ABF) [mm]	F95 % (NO – ABF) [Hz]	Acc-RMS (NO – ABF) [mm/s^2^]	COP-RMS (ABF) [mm]	F95 % (ABF) [Hz]	Acc-RMS (ABF) [mm/s^2^]	COP-RMS %difference	F95% %difference	Acc-RMS %difference
Subj. #1	10.79 (2.84)	0.99 (0.05)	137 (48)	9.57 (1.86)	1.18 (0.16)	118 (13)	-11.2	19.1	-14.1
Subj. #2	9.91 (2.77)	1.20 (0.29)	142 (27)	9.50 (2.26)	1.30 (0.20)	120 (23)	-4.1	8.7	-15.6
Subj. #3	9.21 (2.94)	1.16 (0.14)	121 (23)	8.61 (1.42)	1.37 (0.07)	113 (21)	-6.5	18.0	-7.0
Subj. #4	10.23 (1.50)	1.43 (0.08)	117 (30)	8.80 (1.74)	1.49 (0.12)	100 (12)	-13.9	4.1	-14.6
Subj. #5	8.50 (0.93)	1.49 (0.22)	143 (46)	6.90 (1.35)	1.53 (0.28)	115 (19)	-18.8	2.6	-19.3
Subj. #6	9.62 (1.55)	1.34 (0.30)	126 (43)	7.35 (0.88)	1.34 (0.09)	89 (20)	-23.6	0.0	-29.2
Subj. #7	6.37 (1.48)	1.60 (0.07)	64 (8.3)	5.19 (0.59)	1.94 (0.12)	51 (4.7)	-18.5	20.8	-20.1
Subj. #8	6.08 (1.19)	1.78 (0.25)	48 (6.3)	6.75 (1.41)	1.37 (0.16)	39 (3.8)	10.9	-23.1	-17.3

Average	8.84 (1.75)	1.37 (0.26)	112 (36)	7.83 (1.54)	1.44 (0.15)	93 (31)	-10.7 (10.92)	6.2 (14.4)	-17.2 (6.3)

### Acceleration analysis

Acceleration sensed at trunk level (L5) in AP direction was significantly reduced by ABF. T-test results also revealed significant effects of ABF on Acc-RMS (p = 0.0009). Acc-RMS was reduced by ABF across all subjects, as shown in Table 1 (last column).

Average reduction of Acc-RMS was 17.2% (Figure 2). Columns 3 and 7 of Table 1 also show the subject-by-subject values of Acc-RMS without and with ABF, respectively. The last three subjects were females and showed smaller Acc-RMS, as expected considering their smaller heights [26].

### Stabilogram diffusion analysis

**Figure 4:**
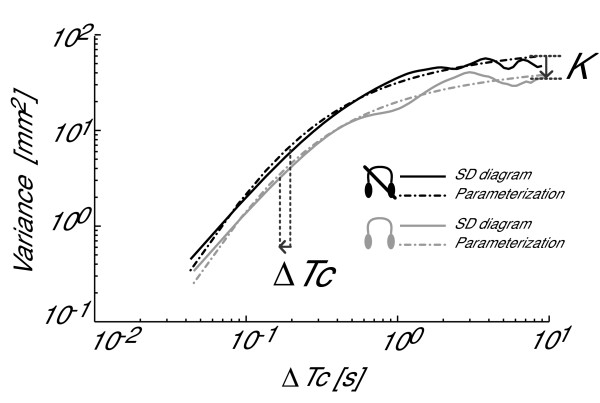
**Effect of ABF on postural control strategy**. SDA diagrams for one representative subject. Two conditions are reported: without ABF (black) and with ABF (gray). The behaviour of K and ΔTc used to parameterize the SDA diagrams is also shown. This figure suggests that, using ABF, subjects decrease the amount of sway by increasing the closed-loop (feedback) posture control.

SDA diagrams plotted from AP COP data, were also significantly influenced by ABF (Figure 4). As a consequence, the parameters K and ΔTc characterizing the SDA diagram were both significantly decreased by ABF (Figure 2). Average K reduction was 9.3% (p = 0.02), whereas average ΔTc reduction was 33.9% (p = 0.018). Table 2 reports the subject-by-subject values of K and ΔTc in both conditions tested. Subject #8 and subject #7 are the only ones who showed a slight increase in K.

**Table 2:** ABF effect on SDA parameters Parameters. K and ΔTc are reported, subject-by-subject, for trials with and without ABF. Percentage differences between these two conditions are also reported. Standard deviations are indicated in parenthesis.

	K (NO-ABF) [mm^2^]	Δtc (NO-ABF) [s]	K (ABF) [mm^2^]	Δtc (ABF) [s]	K % difference	Δtc % difference
Subj. #1	100 (57)	0.42 (0.21)	86 (15)	0.38 (0.17)	-14.6	-9.9
Subj. #2	70 (29)	0.51 (0.31)	66 (20)	0.41 (0.34)	-7.4	-20.5
Subj. #3	75 (41)	0.52 (0.29)	65 (20)	0.29 (0.12)	-13.3	-45.3
Subj. #4	80 (21)	0.81 (0.46)	70 (14)	0.39 (0.14)	-11.1	-52.0
Subj. #5	47 (13)	0.32 (0.08)	39 (10)	0.26 (0.16)	-18.1	-19.7
Subj. #6	64 (12)	0.27 (0.08)	61 (9)	0.20 (0.09)	-5.7	-26.1
Subj. #7	32 (7)	0.17 (0.06)	34 (9)	0.09 (0.01)	6.6	-47.4
Subj. #8	35 (14)	0.29 (0.09)	38 (13)	0.19 (0.06)	5.8	-34.3

Average	63 (23)	0.41 (0.20)	57 (18.5)	0.27 (0.11)	-9.3 (9.2)	-33.9 (15.3)

### Muscle activity analysis

**Figure 5:**
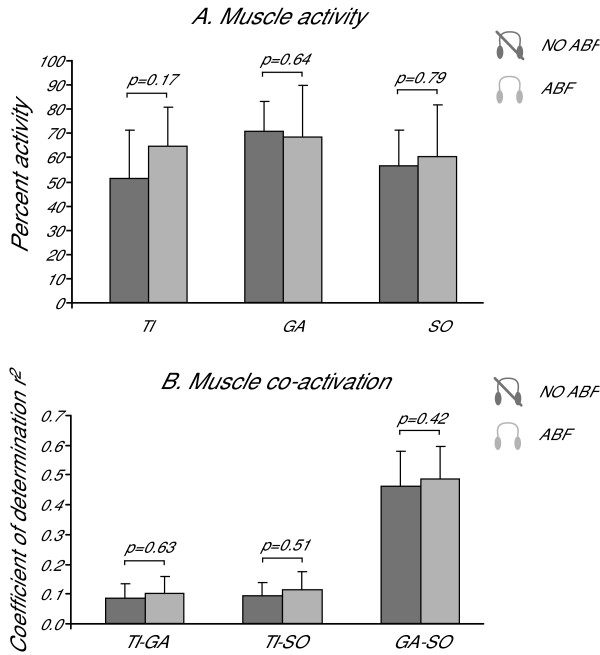
**Effect of ABF on muscle activity**. Estimates of muscle activity levels (Fig. 5A) and muscular co-activation (Fig. 5B) for different pairs of muscles (TI-GA, TI-SO, GA-SO) are shown. Average values are reported for trials with (light gray) and without (dark gray) ABF. Error bars represent standard deviations. As shown in Figure 5A, using ABF does not change significantly the activity of the muscles analyzed (p values from T-Test are reported). This suggests that the major amount of postural corrections induced by ABF does not involve a major average activity of the muscles TI, GA, and SO in the leg. As shown in Figure 5B, using ABF does not change significantly the co-activation between the muscles analyzed (p values from T-Test are reported). This suggests that the major amount of postural corrections induced by ABF does not involve a major co-activation of the muscles TI, GA, and SO in the leg.

Muscle activity of TI, GA, and SO was not influenced by ABF. Overall, the mean activity, expressed as a percentage of the maximal activity recorded from each single muscle across all the trials of a subject, did not change significantly due to ABF (see Figure 5A). TI activity showed a trend toward increasing in trials with ABF (p = 0.17) but this change was particularly clear only for subjects #4 and #7.

Muscle co-activation of ankle agonists-antagonists did not change significantly due to the ABF (see Figure 5B). Co-activation between TI and GA was small both with (r^2 ^= 0.11) and without (r^2 ^= 0.08) ABF. Similarly small was the co-activation between TI and SO with (r^2 ^= 0.14) and without (r^2 ^= 0.09) ABF. As expected, co-activation between the agonists muscles, GA and SO, was instead large (r^2 ^= 0.39 in trials with ABF and r^2 ^= 0.46 in trials without ABF). Figure 5B reports the coefficient of determination r^2^, which indicates the amount of muscular co-activation, for all pairs of muscles analyzed in trials with and without ABF.

## Discussion

Using the proposed ABF device, all healthy subjects included in this study could sway less when standing in a particularly challenging condition, with vision unavailable and somatosensation partly unreliable. All subjects, in fact, reduced their AP Acc-RMS (see Table 1). In this way, subjects were further from their stability limits and, consequently, more stable. Trunk stabilization resulted in smaller corrective torques at the ankles, and hence smaller COP displacements. All but one subjects (Subj. #8) showed a significant decrease in AP COP-RMS (Fig. 2). During ABF, postural corrections in leg muscles were smaller but more frequent in number, as suggested by the increase in F95% of the COP. Future studies involving more sophisticated techniques for the acquisition and analysis of the EMG signals will be needed to validate this hypothesis. This result suggests that ABF may partially substitute for the lack of visual and somatosensory information for postural control by taking the postural control system towards a new steady state associated with a different control strategy.

Examination of the SDA and the EMG activity supported the hypothesis that ABF does not induce an increased stiffness (and hence more co-activation) in leg muscles, but rather helps the brain to actively change to a more feedback-based control over standing posture. Representative SDA diagrams reported in Figure 4 suggest that ABF contributes to a general reduction of both the diffusion coefficient K and the transition time ΔTc. Downward shifts of the SDA diagrams, described by smaller diffusion coefficients, reflect a reduced stochastic activity of the COP, and hence a more tightly regulated control system [16]. Shorter transition times reflect an earlier switching between persistent and anti-persistent behaviors, and hence more prompt reactions to perturbations of the postural control system [27]. In summary, these results support the hypotheses that ABF: 1) increases postural stability in stance, and 2) results in a more prominent role for feedback control over feed-forward control. Hence, the solution proposed by the brain with ABF seems to involve more feedback control for a more stable sway.

Interestingly, our results differ from the results observed by Rougier in quiet stance experiments with visual BF [28]. With visual BF, SDA diagrams only changed some local properties (local slopes) over short or long observation intervals but did not shift significantly, consistent with little, if any, change in K. Furthermore, with visual BF, closed-loop control operated over longer observation-times, suggesting that feed-forward control expanded over feedback control. Such a different behavior between auditory and visual BF may be due to the peculiar, non-redundant role of different senses in multi-sensory integration for the control of posture [29]. Whereas vision provides information about the external environment, it allows predictions of forthcoming events in the scene (feed-forward control) [30]. In contrast, hearing, compared to vision, may be more important for postural reactions to disturbing stimuli (feedback control). This result can also be related to the different processing times required by the central nervous system for visual and auditory stimuli with auditory reaction times significantly faster than visual reaction times. Finally, another factor which may explain the different outcomes of the two BF-studies is the selection of two, different, input variables (COP for visual BF and Acceleration from the trunk for ABF). It is widely accepted that upper- and lower- body segments are controlled separately [31].

Both predictive (feed-forward) and reactive (feedback) control need to be used in order to have an adequate interaction with the environment for postural stability. For this reason, it's hard to determine the relative validity of audio and visual BF. Rather, it may be important, in a rehabilitation setting, to identify which one of the two components of postural control (feed-forward or feedback) needs more reinforcement or substitution in a particular patient, and consequently design an optimised BF treatment.

**Figure 6:**
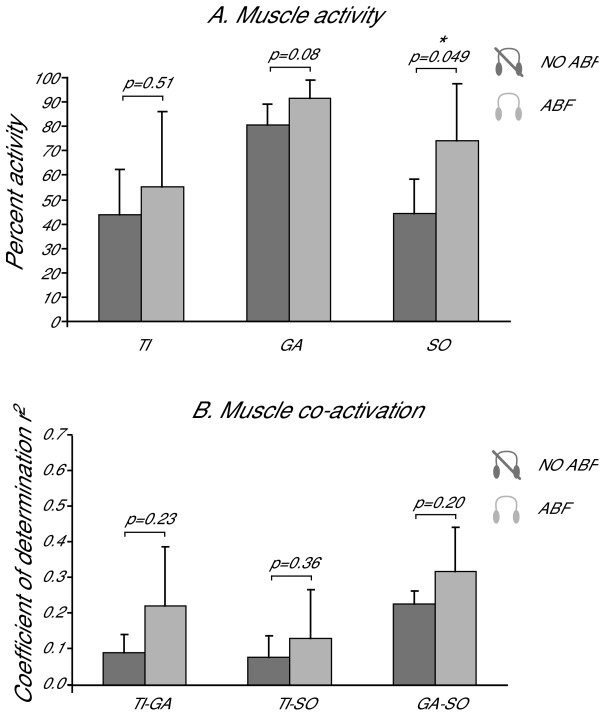
**Muscle activity and co-activation in subject #8**. The antithetic behaviour of subject #8 for muscles activity (Fig. 6B), and for muscles co-activation (Fig. 6A) is shown. Figure 6A reports the estimates of muscular activity for TI, GA, and SO muscle. Average values expressed in percentage are reported for trials with (light gray) and without (dark gray) ABF. Error bars represent standard deviations. The percent activity was calculated taking as one-hundred-percent reference the trial with the highest muscle activation recorded. Even if muscle activity looks higher in trials with ABF for all muscles, only SO activity changed significantly while using ABF (p values from T-Test are reported; since the number of samples is five, it is convenient to report also the powers which were respectively: 0.09, 0.41, 0.53). This suggests that a major amount of activity of the muscles TI, GA, and SO was exercised by this subject while using ABF. Figure 6B reports the estimates of muscular co-activation for different pairs of muscles: TI-GA, TI-SO, and GA-SO. Average values are reported for trials with (light gray) and without (dark gray) ABF. Error bars represent standard deviations. Even if co-activation looks higher in trials with ABF for all couples of muscles while using ABF, muscles co-activation does not change significantly (p values from T-Test are reported; since the number of samples is five it is convenient to report also the powers which were respectively: 0.20, 0.14, 0.23). This suggests that a major amount of co-activation of the muscles TI, GA, and SO was exercised by this subject while using ABF.

The outlying results observed for Subj. #8 need to be discussed individually. This woman in fact did not decrease COP-RMS and K, and did not increase F95%, although, similarly to the other subjects, she decreased Acc-RMS and ΔTc (these changes were consistent across the whole population). Hence, with ABF she actually swayed less and she showed the same increase of feedback control. Nonetheless, either due to her small body size or to a slightly different control scheme, she obtained these goals with a different strategy. Figure 6 reports her muscle activities and co-activations. It can be seen how she generally increases muscle activity with ABF (Figure 6A), in particular with a large increase in the activity of posterior muscles, GA and SO. It should be noted, however, that also the estimated co-activations (Figure 6B) look pretty dissimilar compared with the ones of the other subjects, shown in Figure 5B. Particularly low is the co-activation of agonists muscles GA-SO without ABF, which ABF partly contributes to enlarge. For all these reasons, her postural behavior in the proposed task should be looked as an outlying behavior and more analyses are needed, on a larger population, to assess the real influence of body size or usual control strategies on the responsiveness to ABF.

Many earlier biofeedback systems used audio alarms to notify the user of abnormal values of monitored parameters (e.g. [32]). The present ABF system is novel in the use of nonlinear coding functions and in the customization of these functions for each subject and task [9]. Although the current ABF system may interfere with use of hearing for communication, it may be quite useful during the rehabilitation and training process. Plans are underway to improve the current ABF system by making it wireless for increased portability and equipping it with a communication module for remote control, recording, and monitoring. Different sonification procedures will also be tested and compared in a near future. Specifically, 3-D generated sound with a HRTF (Head Related Transfer Function) or immersive sound may be even more effective signals for improving stance balance.

## Conclusion

We have investigated the attributes of a portable instrument that feeds back trunk acceleration in order to help subjects to reduce their postural sway during stance. The instrument meets requirements for an adequate biofeedback system that may find interesting applications not only as a rehabilitation device in the clinic, but also in the home care setting, and when doing community mobility training outside the traditional clinical setting. In fact, it has appropriate bandwidth and sensitivity, smoothness and delay of the acoustic signal generator, as well as portability. Acoustic information related to trunk movement allowed subjects in the present experiment to increase postural stability when sensory information from both vision and the surface were compromised by eye closure and stance on foam. We provided evidence that the balance improvement was not a stiffening at the ankle, but rather the brain actively adapted its control strategy over standing posture with more feedback-based control.

## List of abbreviations

ABF = audio biofeedback

Acc-RMS = root mean square of the acceleration

AP = anterior-posterior

BF = biofeedback

COP = center of pressure

COP-RMS = root mean square of the COP

EMG = electromyography

F95% = frequency comprising the 95% of the power

GA = gastrocnemius

ML = medial-lateral

SDA = stabilogram diffusion analysis

SO = soleus

TI = tibialis

## Competing interests

The authors of this paper declare that they applied for a patent (patent application PCT/IB2004/001679) concerning the audio-biofeedback device used for the study described in this paper. The patent is property of the University of Bologna and of the Oregon Health & Science University.
